# Stimulatory Effects of Polysaccharide Fraction from *Solanum nigrum* on RAW 264.7 Murine Macrophage Cells

**DOI:** 10.1371/journal.pone.0108988

**Published:** 2014-10-09

**Authors:** Faizan Naeem Razali, Amirah Ismail, Nurhayati Zainal Abidin, Adawiyah Suriza Shuib

**Affiliations:** 1 Institute of Biological Sciences, Faculty of Science, University of Malaya, Kuala Lumpur, Malaysia; 2 University of Malaya Centre for Proteomics Research, Faculty of Medicine, University of Malaya, Kuala Lumpur, Malaysia; Dasman Diabetes Institute, Kuwait

## Abstract

The polysaccharide fraction from *Solanum nigrum* Linne has been shown to have antitumor activity by enhancing the CD4^+^/CD8^+^ ratio of the T-lymphocyte subpopulation. In this study, we analyzed a polysaccharide extract of *S. nigrum* to determine its modulating effects on RAW 264.7 murine macrophage cells since macrophages play a key role in inducing both innate and adaptive immune responses. Crude polysaccharide was extracted from the stem of *S. nigrum* and subjected to ion-exchange chromatography to partially purify the extract. Five polysaccharide fractions were then subjected to a cytotoxicity assay and a nitric oxide production assay. To further analyze the ability of the fractionated polysaccharide extract to activate macrophages, the phagocytosis activity and cytokine production were also measured. The polysaccharide fractions were not cytotoxic, but all of the fractions induced nitric oxide in RAW 264.7 cells. Of the five fractions tested, SN-ppF3 was the least toxic and also induced the greatest amount of nitric oxide, which was comparable to the inducible nitric oxide synthase expression detected in the cell lysate. This fraction also significantly induced phagocytosis activity and stimulated the production of tumor necrosis factor-α and interleukin-6. Our study showed that fraction SN-ppF3 could classically activate macrophages. Macrophage induction may be the manner in which polysaccharides from *S. nigrum* are able to prevent tumor growth.

## Introduction

In recent decades, the engineering and production of increasingly efficient chemotherapies has become possible. Despite this, there continues to be a need for additional targeted therapies with fewer side effects and greater specificity. Among the newer options for treatment is the possibility to modulate the immune response within the patient, making the patient's own immune system more capable of eliminating cancer cells.

Studies have shown that a variety of polysaccharides can act as immunomodulators, helping to stimulate the immune system so that it can eliminate tumors and foreign invaders more effectively. Unlike bacterial polysaccharides, polysaccharides derived from higher plants are typically nontoxic and often do not cause side effects [1], which make them promising potential candidates for cancer therapy. Plant polysaccharides are able to activate macrophages, which in turn increases nitric oxide (NO), cytokines, chemokines, inducible nitric oxide synthase (iNOS), and phagocytosis *in vitro* [1]. In addition to macrophage activation, some plant polysaccharides stimulate the activities of natural killer cells, which are cells of the innate immune system responsive to viral invasion as well as the early formation of tumor cells [2], and B cells, which are members of the adaptive immune system that launch antibody-mediated attacks on antigens [3]. The antitumor activity of polysaccharides has been observed in cancer-bearing ICR and S180 mice treated with *Dendrobium denneanum* and *Actinidia eriantha* extracts, respectively [4], [5]. Tumor regression and enhanced immune response have been observed in these animals.

The herbal plant *Solanum nigrum* is widely distributed throughout the world, extending from tropical to temperate regions [6]. Because of the folkloric belief that it imparts numerous benefits, including curing cancer, it has been studied extensively [7]. Studies done by several researchers showed that crude extract from *S. nigrum* suppresses free radical–mediated DNA damage [8], induces necrosis in SC-M1 stomach cancer cells [9], inhibits 12-*O*-tetradeca-noylphorbol-13-acetate-induced tumor promotion in MCF-7 breast cancer cells [10], and has antineoplastic activity against several human tumor cell lines [11]. An in-depth study showed that the polysaccharide from *S. nigrum* Linne has an antitumor effect on tumor-bearing Kunming mice by significantly enhancing the CD4^+^/CD8^+^ ratio of the T-lymphocyte subpopulation [12]. In our study, the *S. nigrum* polysaccharide extract was further evaluated to examine how it modulates immune responses and to uncover a potential mechanism for this activity. Because macrophages play an important role in inducing both innate and adaptive immune responses, the effects of the *S. nigrum* extract on the RAW 264.7 mouse macrophage cell line were evaluated by measuring NO production, tumor necrosis factor (TNF)-α and interleukin (IL)-6 secretion, and the rate of macrophage phagocytosis.

## Materials and Methods

### Reagents and chemicals

N-1-napthylethylenediamine dihydrochloride and sulfanilamide were purchased from Acros Organics (Geel, Belgium). Dimethyl sulfoxide, MTT [3-(4,5-dimethylthiazol-2-yl)-2,5-diphenyl-tetrazolium bromide], Dulbecco's modified Eagle medium, E-TOXATE™ kit, monosaccharide standards (arabinose, xylose, fucose, rhamnose, galactose and mannose) and lipopolysaccharide (LPS) from *Escherichia coli* 055:B5 were obtained from Sigma-Aldrich (St. Louis, MO, USA). Ethanol, petroleum ether, and D-(+)-glucose were purchased from Merck (Darmstadt, Germany). Fetal bovine serum, penicillin/streptomycin, and amphotericin B were from PAA Laboratories (Cölbe, Germany). The Phagocytosis Assay Kit IgG fluorescein isothiocyanate (FITC) and the iNOS polyclonal antibody were purchased from Cayman Chemical (Ann Arbor, MI, USA). The TNF-α mouse ELISA kit and the IL-6 mouse ELISA kit were obtained from Abcam (Cambridge, UK). Diethylaminoethyl cellulose pre-swollen ion exchanger was purchased from Whatman (Maidstone, England). A 2-D protein extraction buffer was purchased from GE Healthcare (Piscataway, NJ, USA). β-actin polyclonal antibodies were purchased from BioVision Research Instrument (Milpitas, CA, USA). Donkey anti-rabbit IgG was obtained from Abnova (Taipei City, Taiwan).

### Plant collection and sample preparation

Fresh whole *S. nigrum* L. *nigrum* plants were purchased from the local market located at Lembah Pantai, Kuala Lumpur, in February 2012. The plants were identified by Dr. Sugumaran Manickam of Institute of Biological Sciences, Faculty of Science, University of Malaya, Kuala Lumpur, Malaysia, and a voucher specimen was deposited at the herbarium of the Institute of Biological Sciences, Faculty of Science, University of Malaya, Kuala Lumpur, Malaysia (herbarium number: KLU 47872). *S. nigrum* polysaccharides were extracted according to described method [13]. Briefly, the stems of *S. nigrum* were washed, and then dried at 45°C, and ground to a fine powder. To extract the polysaccharides, 333.7 g of the *S. nigrum* powder was refluxed using a Soxhlet apparatus with 2 L of petroleum ether (60°C–80°C) to remove waxes, fats, and volatile oils from the sample [14]. Then the sample was refluxed again with 2 L of 80% ethanol to remove monosaccharides and oligosaccharides. The residue was then boiled in 2 L of 95°C water for 5 hours before it was filtered through Whatman no. 3 filter paper. The filtrate was then purified in equal volume of 70% ethanol, allowing the polysaccharide to precipitate overnight at 4°C. The precipitate was collected by centrifugation at 4,000×*g* in 4°C for 20 minutes and then washed twice with 95% ethanol. The *S. nigrum* crude polysaccharide pellet was allowed to dry in a desiccator for 7 days before it was stored at 4°C for further use.

### Ion-exchange chromatography

*S. nigrum* crude polysaccharide (300 mg) was subjected to purification by a diethylaminoethyl cellulose column (ø20 mm×250 mm), which was equilibrated with 5 mM sodium phosphate buffer, pH 7.4. The polysaccharide component was eluted with a linear gradient of 0–1.5 M sodium chloride in 5 mM sodium phosphate buffer. Two-hundred fractions were collected in 5 mL aliquots using a fraction collector. The polysaccharide content of each fraction was determined by the phenol-sulfuric acid method described below. Based on the chromatographic profile, the apex fractions of the five peaks observed, were pooled and dialyzed four times using SnakeSkin Pleated Dialysis Tubing (Thermo Scientific, Tewksbury, MA, USA) against 1 L distilled water. Finally, the fractions were freeze-dried and stored at −20°C.

### Measurement of carbohydrate and protein content

Total carbohydrate content was estimated using phenol-sulfuric acid method [15]. Briefly, 50 µL of a 100 mg/mL sample was pipetted into a 96-well plate (Orange Scientific, Braine-l'Alleud, Belgium); 150 µL of absolute sulfuric acid was added into each well, followed immediately by 30 µL of a 5% w/v phenol in distilled water. The plate was heated in a 95°C water bath for 10 minutes. The optical density of the samples (490 nm) was measured by a Multiskan Go microplate spectrophotometer (Thermo Scientific). The carbohydrate concentration was determined using the standard curve of D-glucose [16]. The protein content of the samples was quantified using Bradford Protein Quantification Kit (Bio-Rad, Hercules, CA, USA).

### Cell line and culture medium

The mouse macrophage cell line, RAW 264.7, was purchased from the American Type Culture Collection (Manassas, VA, USA) and was maintained in Dulbecco's modified Eagle medium supplemented with 10% fetal bovine serum, 2% of 100× penicillin/streptomycin and 1% of 100× amphotericin B. The cells were cultured in a humidified 5% CO_2_ and 100% humidified incubator (Shel Lab, Cornelius, OR, USA) at 37°C.

### Cytotoxicity evaluation

Described originally by Mosmann (1983), an MTT cell proliferation assay was used to evaluate the cytotoxicity of the *S. nigrum* polysaccharide fractions on RAW 264.7 cells [17]. Briefly, RAW 264.7 cells (5×10^5^ cells/mL) were seeded in a 96-well culture plate (Orange Scientific) and incubated for 24 hours. The cells were then treated with polysaccharide samples at 0, 1.56, 3.125, 6.25, 12.5, 25, 50, and 100 µg/mL for 72 hours. To observe cell viability, a 20-µL aliquot of MTT solution was added to each well and incubated for another 4 hours. The purple formazan that developed was diluted with 200 µL dimethyl sulfoxide, and the absorbance values at 570 nm and 690 nm were measured by a Multiskan Go microplate spectrophotometer. The cytotoxicity for each sample was expressed as the IC_50_, which is the concentration of each extract that reduces cell survival by 50% when compared with the untreated control.

### Measurement of NO production

For the NO production assay, RAW 264.7 cells (5×10^5^ cells/mL) were seeded into a 96-well culture plate and treated with the polysaccharide fractions at 12.5, 25, 50, and 100 µg/mL for 24 hours. LPS at 100 µg/mL was used as a positive control. Griess reagent (1% sulfanilamide, 0.1% N-1-napthylethylenediamine dihydrochloride, and 2% phosphoric acid) was added to each well, and the plate was incubated in the dark at room temperature for 10 minutes. Absorbance at 520 nm was measured by a Multiskan Go microplate spectrophotometer.

### iNOS detection by Western blotting

On the basis of NO production assay, the *S. nigrum* polysaccharide fraction SN-ppF3 was chosen for the iNOS detection analysis. To assess polysaccharide-induced iNOS expression, RAW 264.7 cells (5×10^5^ cells/mL) were seeded in a six-well cell culture plate and treated with polysaccharide fraction SN-ppF3 at 100 µg/mL for 24 hours. LPS was used as a positive control at the same concentration. Cells were harvested using a scraper, washed with 10 mM Tris-sucrose buffer (pH 7.0), and centrifuged at 1,000×*g* for 5 minutes. The cells were lysed with 2 mL of 2-D protein extraction buffer III (GE Healthcare) and pipetted several times on ice. The cell lysate was collected by centrifuging the lysed cells at 42,000×*g* at 4°C for 10 minutes. The cell lysate was resolved by 10% SDS-PAGE (90 V for 90 minutes), and the protein was transferred onto a nitrocellulose membrane for 1 hour. The membrane was blocked with 3% w/v gelatin in Tris-buffered saline-Tween 20 buffer (pH 7.4) for 1 hour [18]. The membrane was cut into two pieces based on the molecular weight markers, in which the piece containing higher molecular weight proteins was incubated with anti–mouse iNOS and the piece containing lower molecular weight proteins was incubated with anti–mouse β-actin overnight at room temperature. After washing three times (5 minutes each wash) with Tris-buffered saline-Tween 20 buffer (pH 7.4), the membranes were incubated with horseradish peroxidase–conjugated anti-rabbit secondary antibody for 1 hour at room temperature. Following the final wash, the membranes were developed by incubating in 3,3′-diaminobenzidine substrate (Thermo Scientific) in the dark for 5 minutes. The membrane was then washed with distilled water and scanned. The intensity of each band was analyzed using ImageJ 1.48a (National Institutes of Health, Bethesda, MD, USA).

### Phagocytosis

To activate the cells for phagocytosis, of RAW 264.7 cells (5×10^5^ cells/mL) were seeded in a six-well plate (Orange Scientific) and incubated for 24 hours. Then, 100 µg/mL of SN-ppF3 was added to each well and incubated for another 24 hours. LPS was added at 100 µg/mL as a positive control. To quantify the phagocytosis activity, a phagocytosis assay kit (IgG FITC) was used. Briefly, the cells were mixed with 100 µL of latex beads conjugated to rabbit IgG-FITC and incubated for 24 hours. The cells were then centrifuged for 5 minutes at 400×*g* at room temperature. The supernatant was removed and the cells were washed two times with 1 mL of assay buffer. Finally, the cells were suspended in 500 µL of assay buffer and immediately analyzed using a BD FACSCanto II flow cytometer (BD Biosciences, San Jose, CA, USA). The experimental results were compared to both the untreated (negative control) and LPS-treated (positive control).

### TNF-α and IL-6 production

In this assay, the cells were prepared as in the phagocytosis activity preparation section. After 24-hour incubation with the SN-ppF3 fraction or LPS, the culture media in each well was collected and the presence of TNF-α and IL-6 were assayed using ELISA kits. All reagents and solutions required for this assay were provided in the ELISA kit. Culture media (100 µL) was pipetted into a 96-well microplate coated with either anti–mouse TNF-α or anti-mouse IL-6 and was incubated overnight at 4°C. The medium was discarded, and the wells were washed four times with 300 µL washing buffer. Biotinylated anti–mouse TNF-α or IL-6 antibody (100 µL) was added into each well, and the plate was incubated for 1 hour at room temperature with gentle shaking. The solution was discarded, and the wells were washed before 100 µL of horseradish peroxidase-streptavidin solution was added into each well. The plate was incubated for 45 minutes at room temperature with gentle shaking. After the final wash, 100 µL TMB One-Step Substrate Reagent was added to each well, and the plate was incubated for another 30 minutes in the dark with gentle shaking. Stop solution (50 µL) was added into each well to stop the color development, and the absorbance at 450 nm was immediately measured by a Multiskan Go microplate spectrophotometer. The standard curve for the recombinant TNF-α and IL-6 proteins was used to determine the concentration of the cytokines.

### Endotoxin test

The presence of endotoxin in the sample was detected by using qualitative commercial Limulus amebocyte lysate (LAL) E-TOXATE™ kit (Sigma Aldrich, St. Louis, MO, USA). This test was carried out according to manufacturer's protocol. Briefly, 100 µL of E-TOXATE Reagent was added into 100 µL of 100 µg/mL SN-ppF3, 100 µL of endotoxin standard and 100 µL of E-TOXATE water. The mixture were gently mixed, covered with foil and incubated in 37°C undisturbed for 1 hour. The incubation period was followed by observing any evidence of gelation by inverting the tubes 180° gently. The presence of endotoxin contamination will result the formation of hard gel in the tubes which permit complete inversion without any disruption.

### Monosaccharide composition analysis

Twenty milligrams of SN-ppF3 was hydrolyzed with 2 mL of 4 M trifluoroacetic acid (TFA) (Sigma, St. Louis, MO, USA) in a glass test tube for 5 hours at 95°C water bath. The hydrolysate was then evaporate with a stream of nitrogen gas at 80°C, washed with absolute methanol and redissolved with 1 mL of 75% Acetonitrile (ACN).It was filtered through 0.45 µM syringe filter and separated using Agilent Carbohydrate Analysis Column on Agilent HPLC 1260 (Agilent Technologies, Santa Clara CA, USA), equipped with refractive index detector. The column and detector temperature were set at 30°C and sample was eluted isocratically with 75% ACN at a flow rate of 1.4 mL/min. The possible identity of monosaccharides in the samples was determined by comparing the retention time of the peaks with those of standards (rhamnose, fucose, xylose, arabinose, glucose, galactose and mannose).

### Statistical analysis

The MTT assay, nitric production assay, phagocytosis analysis, and cytokine production measurement were performed no fewer than three times each. The data were subjected to one-way analysis of variance (ANOVA), with the significant difference between the means determined by Duncan's multiple-range test at 95% significant different (*p*<0.05) using SPSS 17.0 Statistics software (IBM Corporation, Endicott, NY, USA). The graphs depicting the results of ion-exchange chromatography and the NO concentration, as well as all of the standard curves were constructed using GraphPad Prism 5 software (GraphPad Software Inc, CA, USA).

## Results and Discussion

### Ion-exchange chromatography of *S. nigrum* crude extract

**Figure 1:**
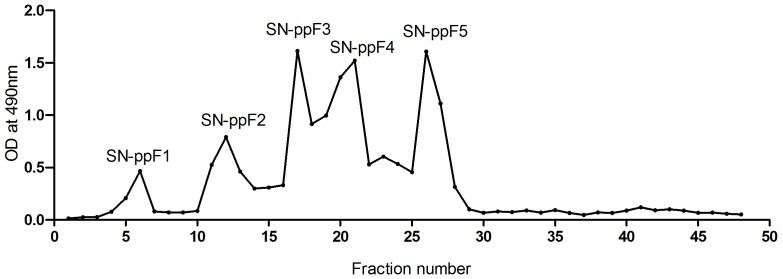
Ion-exchange chromatography profile of *S. nigrum* crude polysaccharide extract on a diethylaminoethyl cellulose column.

The *S. nigrum* crude polysaccharide extract was subjected to ion-exchange chromatography. The plotted chromatography profile (Figure 1) indicates that the crude extract resolved into five peaks high in sugar residues within the first 30 fractions. There was no peak observed beyond fraction 30. These peaks were then labeled as SN-ppF1, SN-ppF2, SN-ppF3, SN-ppF4, and SN-ppF5. Li et al. (2010) performed ion-exchange chromatography on *S. nigrum* crude polysaccharide extract; however, the profile obtained in our study was not comparable with what was reported by the Li group as they obtained three major peaks [12]. This could be explained by differences in the plants used: (1) the extract in our study was prepared from the stem of *S. nigrum* instead of the whole plant and (2) the plants were grown in different locations with differing climates. Li's group obtained their sample from Taihang Mountain in the Hebei province located in China, whereas the sample for this study was grown by farmers at a lower ground in a tropical climate, located in Raub, Pahang, Malaysia. It has been reported that differences in environment, season, and climate affect plant composition [19]–[21].

### Carbohydrate and protein content in *S. nigrum* fractions

The five polysaccharide peaks were subjected to carbohydrate and protein content analysis using the phenol-sulfuric acid method and the Bradford method, respectively. The carbohydrate and protein contents are summarized in Table 1. SN-ppF1 had the highest carbohydrate content of the five peaks (98.0%) followed by SN-ppF4, SN-ppF3, SN-ppF2, and then SN-ppF5. Thus, the fractions were not purely polysaccharide. Each fraction contained less than 1% of its total weight in protein. Therefore, the fractions may not contain glycoprotein, given the very low protein content observed. Beside polysaccharides, several studies have shown some glycoproteins also have immunomodulatory properties [22], [23].

**Table 1:** Carbohydrate and protein content in 100 mg/mL of *S. nigrum* polysaccharide fractions.

Sample	Content (mg/mL)	
	Carbohydrate	Protein
SN-ppF1	97.98±1.6^a^	0.96±0.03^z^
SN-ppF2	74.40±1.2^c^	0.37±0.02^y^
SN-ppF3	79.62±2.8^b^	0.11±0.05^w^
SN-ppF4	94.88±2.2^a^	0.15±0.05^x^
SN-ppF5	74.18±2.4^c^	0.05±0.03^w^

Values expressed are mean ± standard deviation (*n* = 3). Different uppercased letters in each column indicate a significant difference at *p*<0.05.

### *In vitro* cytotoxic analysis of *S. nigrum* polysaccharide fractions

The MTT assay was used to detect cytotoxic effects of the *S. nigrum* polysaccharide fractions on RAW 264.7 cells. However, even at the maximum concentration of 100 µg/mL and after 72 hours incubation period, the IC_50_ values could not be determined. According to the U.S. National Cancer Institute plant screening program, the IC_50_ value for a crude plant extract considered to be cytotoxic is less than 20 µg/mL upon incubation for 48**–**72 hours [24]. Therefore, it could be concluded that none of the polysaccharide fractions had cytotoxic effects on RAW 264.7 cells after the 72 hours incubation period (Table 2). However, looking at the killing percentage at the maximum concentration tested (100 µg/mL), SN-ppF1 showed the highest cytotoxic effect, with 48.7%±3.7 of cells dying, followed by SN-ppF5, SN-ppF4, SN-ppF2, and then SN-ppF3. Among all of the fractions, SN-ppF3 had the lowest cytotoxic effect, with 28.5%±2.6 of cells dying after 72 hours treated period. This finding supports the documented statement that higher plant polysaccharide extracts are mostly nontoxic [1].

**Table 2:** Cytotoxic activity (IC_50_ value) of *S. nigrum* polysaccharide fractions against RAW 264.7 cell line and killing percentage at maximum concentration.

Polysaccharide fractions	IC_50_ value (µg/mL)	Killing percentage at 100 µg/mL
SN-ppF1	>100	48.7±3.7^c^
SN-ppF2	>100	38.8±4.4^c^
SN-ppF3	>100	28.5±2.6^b^
SN-ppF4	>100	34.7±10.7^c^
SN-ppF5	>100	41.2±5.4^c^
Non-treated**	>100	0.0±0.01^a^

**Positive control. Values are expressed as mean ± standard deviation (*n* = 3). Different letters (a–c) indicate a significant difference at *p*<0.05.

### NO production

**Figure 2:**
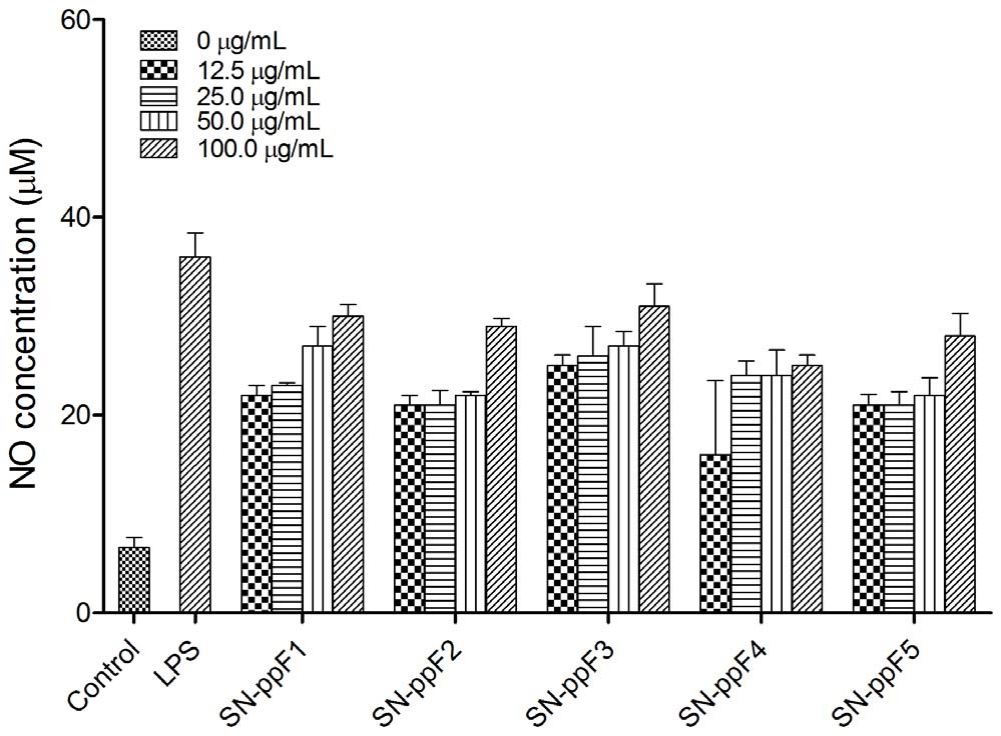
Production of nitric oxide by RAW 264.7 cells treated with polysaccharide fractions SN-ppF1–F5.

One of the killing mechanisms of an activated macrophage is the production of NO, which works in combination with hydrogen peroxide or superoxides. Peroxynitrite radicals produced from this reaction can kill phagocytosed microbes in the macrophage. Besides eliminating microbes, NO is also considered to be one of the most important mediators directly involved in tumor-cell killing [25]. Thus, a cell's NO production can be used as a proxy for the polysaccharide-based activation of macrophages. In this study, when RAW 264.7 cells were treated with *S. nigrum* polysaccharide fractions, NO production was detected (*p*<0.05), suggesting that the polysaccharides was able to induce NO production by macrophage in a dose-dependent manner (Figure 2). To our knowledge, the production of NO induced by polysaccharide fractions of *S. nigrum* or other *Solanum* species has not been reported. Although the NO production was significantly lower than that produced by LPS, the amount was comparable to NO production from 100 µg/mL Chansong-I mushroom β-glucan [26], red ginseng acidic crude polysaccharide [27] and Jew's ear purified polysaccharide [28]. Since SN-ppF3 induced the highest NO production, it was chosen for the next subsequence macrophage activation assays. Besides, SN-ppF3 was the least toxic as compared to the other fractions. Furthermore, when endotoxin test was carried out on this fraction, no endotoxin was detected.

### iNOS detection by western blot

**Figure 3:**
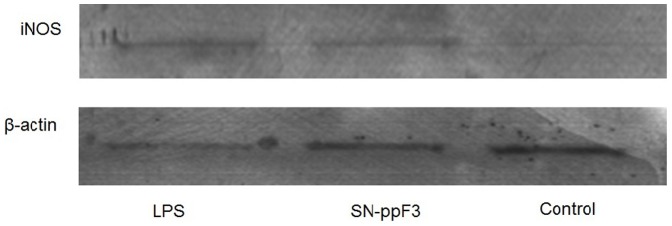
Western blot analysis for detection of iNOS and β-actin in polysaccharide-treated RAW 264.7 cells.

To validate the NO data, the presence of iNOS in the cell lysate of polysaccharide-treated RAW 264.7 cells was determined. NO is synthesized by iNOS, which is absent in resting macrophages. The production of the enzyme can be induced by cytokines such as TNF-α, IL-1β, and IFN-γ (interferon) [29], released by the host. Microbial products such as LPS can directly induce the synthesis of iNOS by binding to CD14, which in turn activates the signaling cascade through Toll-like receptor 4 [30]. iNOS was detected in both LPS- and SN-ppF3-treated cells but not in untreated cells (Figure 3). Semi-quantitative analysis was carried out by dividing the iNOS intensity value by that of β-actin, giving a ratio of 1.69 for LPS-treated cells and 1.39 for SN-ppF3-treated cells. The ratio for the control was 0 because its iNOS level could not be detected. Thus, the expression of iNOS was a reasonable proxy for NO production.

### Phagocytosis

**Figure 4:**
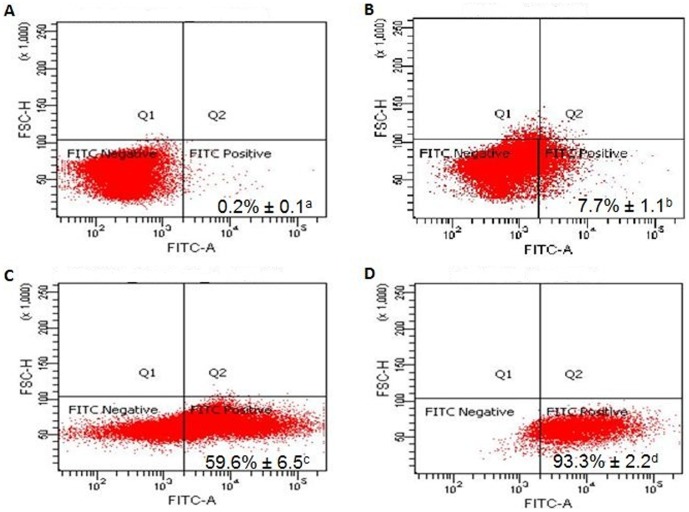
Flow cytometry analysis of IgG-coated bead phagocytosis activity. (A) Untreated cells without latex beads. (B) Untreated cells incubated with latex beads. (C) SN-ppF3-treated cells incubated with latex beads. (D) LPS-treated cells incubated with latex beads. The percentage of cells that phagocytosed beads is indicated. Values are expressed as the mean ± standard deviation (*n* = 3). Different superscript letters (a–d) indicate statistically significant differences (*p*<0.05).

One of the main functions of macrophages is phagocytosis, which is characterized by an engulfment of large particles with diameters of more than 0.5 µm. The process begins with the binding of the particles via receptors on the surface of the macrophage, leading to the formation of a phagosome and eventually to the death of the invading cell or the degradation of the foreign particle. The ability of the activated RAW 247.6 cells to phagocytose foreign particles was tested using fluorescent IgG-coated latex beads. Flow cytometry analysis showed that, without any polysaccharide treatment, only 7.7% of the cells phagocytosed the beads (Figure 4). When the cells were treated with 100 µg/mL of SN-ppF3, the percentage increased to 59.6%. However, LPS gave the highest reading, showing 93.3% of the cells exposed to LPS having phagocytosed the beads. Thus, the phagocytosis activity of the SN-ppF3-treated cells was significantly higher than that of untreated cells but significantly lower than that of LPS-treated cells. Although SN-ppF3 was not as high as LPS in inducing phagocytosis, it is possible that SN-ppF3 and LPS induced the phagocytic activity through similar mechanisms, causing similar biological effects in RAW 264.7 cells. The ability of LPS to induce the immune complex clearance through the Fc gamma receptor is well documented. LPS does not affect the number of Fc gamma receptors expressed on macrophages. Instead, the exposure to LPS causes marked induction of CD11b/CD18, which is the Fc gamma receptor co-transducer [31]. SN-ppF3 might not bind to the same receptor as LPS. However, macrophages may bind to plant polysaccharides specifically through receptors such as Toll-like receptor 4, CD14, CD11b/CD18, scavenger receptor, dectin-1, and mannose receptor, which leads to cytokine/chemokine production, reactive oxygen species production, and cell proliferation [1].

### Effects of SN-ppF3 on cytokine production

Cytokine production is often used as a measure of macrophage activation. The type of cytokine produced will determine the effector function of the macrophage. In a classically activated macrophage, TNF-α along with IFN-γ activate the macrophage, which then starts to produce pro-inflammatory cytokines such as IL-1, IL-6, and IL-23 [32]. To elucidate whether the polysaccharide fraction could classically activate the macrophages, we measured the amount of TNF-α and IL-6 secreted by RAW 264.7 cells. Upon activation by microbes, macrophages secrete TNF-α, which further activates the macrophages in a positive feedback loop, causing a more efficient attack on the foreign invaders. LPS was the most potent signal to induce the production of TNF-α by macrophages. In this study, after 24 hours of incubation with LPS, ∼14.37 ng/mL of TNF-α was secreted into the cell culture media (Table 3). The TNF-α concentration after incubation with SN-ppF3 was ∼13.46 ng/mL. These two values were significantly higher than the value in untreated cells.

**Table 3:** Concentration of TNF-α and IL-6 produced by RAW 264.7 cells treated with SN-ppF3.

Samples	Cytokines production (ng/mL)	
	TNF-α	IL-6
Non-treated**	0.639±0.037^a^	1.964±0.024^x^
SN-ppF3	13.461±0.207^b^	4.051±0.053^y^
LPS*	14.370±0.533^c^	4.341±0.106^z^

*Positive and **negative controls. Results expressed were mean ± standard deviation (*n* = 3). Different letters a–c and x**–**z indicate significant different for TNF-α and IL-6 columns respectively at *p*<0.05.

In response to cytokines such as TNF-α and IL-4, macrophages secrete IL-6. In this study, ∼4.34 ng/mL of IL-6 was secreted into the culture media after 24 hours of incubation with LPS. Comparably, SN-ppF3 caused the production of ∼4.05 ng/mL of IL-6, which was significantly higher than that produced in untreated cells. IL-6 is a cytokine with many roles important for both innate and adaptive immune responses. In the innate immune response, IL-6 plays a role in inducing inflammation, whereas in the adaptive immune response, it is needed by B cells for antibody production and for T cell proliferation and differentiation. Study showed that *S. nigrum* polysaccharide increases the survival of lymphocytes by inhibiting apoptosis [12]. It is possible that this effect is not directly due to the polysaccharide fraction, but is instead elicited by the cytokines produced by innate immune cells, which act on the lymphocytes.

### Monosaccharide composition analysis

The ability of a polysaccharide to induce immunomodulation depends highly on its composition, structure and size. Therefore, a preliminary analysis was carried out to characterize the neutral monosaccharide constituent of SN-ppF3 by hydrolyzing it with TFA at 95°C, At least three neutral monosaccharide were detected, which were rhamnose, glucose and galactose with the molar ratio of 1.00∶0.92∶0.86 (Table 4). However, fucose, xylose, arabinose and mannose were not detected. Thus it is possible that SN-ppF3 contained either rhamnogalacturonan, homogalacturonan or rhamnose hexose types of pectic polysaccharide were presented [33]. The immunomodulatory effect was shown in previous documented study that identified rhamnogalacturonan type of polysaccharide isolated from leaves of *Panax ginseng* C. A. Mayer [34] able to elevate immune complex activity of macrophage by enhancing the expression of Fc receptor on macrophage surface [35]. Similar monosaccharide composition was also describes for tea polysaccharide [36]. However, considering other structural factors, including possible protein-polysaccharide complex which may induce the activity [37], more in-depth study need to carried out in determining the structure and how it interacts with macrophage.

**Table 4:** Neutral monosaccharides composition of SN-ppF3.

Neutral monosaccharides composition (Molar)						
Rhamnose	Fucose	Arabinose	Xylose	Glucose	Galactose	Mannose
8.86	n.d.	n.d.	n.d.	8.13	7.66	n.d.

*n.d. is labeled as not detected.

## Conclusions

In the present study, five polysaccharide fractions were obtained from *S. nigrum*. SN-ppF3 was shown to have no cytotoxic effect in RAW 264.7 cells. Furthermore, incubation with 100 µg/mL of SN-ppF3 generated higher NO compared to the other fractions, increased the phagocytosis activity, and promoted the secretion of TNF-α and IL-6 in RAW 264.7 cells. Thus, the polysaccharide fraction was able to activate macrophages, which may partially explain how polysaccharide from *S. nigrum* imparts the antitumor activity observed *in vivo* [12].
